# Optimizing Lentiviral Vector Production: Insights Into PiggyBac Transposase and Concatemeric Array Strategies

**DOI:** 10.1002/biot.70135

**Published:** 2025-10-06

**Authors:** Jona Röscheise, Maximilian Klimpel, Janina Hoffman, Vathsalya Pabbathi, Herbert Dersch, Parameswari Govindarajan, Holger Laux, Kerstin Otte

**Affiliations:** ^1^ Institute of Applied Biotechnology Biberach University of Applied Sciences Biberach Germany; ^2^ Department of Gene Therapy University Ulm Ulm Germany; ^3^ Biopharmaceutical Product Development CSL Behring Innovation GmbH Marburg Germany

## Abstract

Lentiviral vectors (LVVs) are essential tools in gene and cell therapy due to their ability to transduce both dividing and non‐dividing cells. Conventional production by transient plasmid co‐transfection is variable, costly, and difficult to scale, prompting development of stable producer cell lines. Historically, the GPRTG cell line has been generated using concatemeric‐array integration, which requires high DNA input, complex workflows, and can cause genetic instability. To address these limitations, we evaluated a transposase‐mediated integration strategy. Compared with the concatemeric‐array method, transposase‐based integration enabled faster recovery after selection with only a mild viability crisis and required substantially less DNA. This approach generated highly diverse and heterogeneous producer pools, providing a strong basis for subsequent clonal selection. During LVV production, both methods maintained comparable cell growth stability. However, concatemeric‐derived pools exhibited greater variability in recovery kinetics, viable cell density, and LVV titers, despite achieving the highest maximum titers overall. In contrast, transposase‐mediated pools showed more consistent performance, supporting their reliability for large‐scale applications. In summary, transposase‐based integration offers a robust and scalable alternative to concatemeric‐array methods for generating stable LVV producer cell lines, with significant potential to streamline LVV manufacturing for gene therapy.

## Graphical Abstract and Lay Summary

Transposase‐mediated integration provides a robust alternative for generating stable lentiviral vector producer cell lines. While yielding slightly lower maximum titers than concatemeric‐array methods, it requires less DNA, accelerates recovery, and delivers more consistent performance, supporting scalable LVV manufacturing.

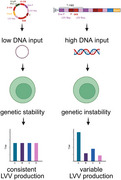

## 1 Introduction

Lentiviruses, a subfamily of retroviruses, are a valuable tool for gene therapy applications, as they can efficiently infect both dividing and non‐dividing cells by integrating their reverse‐transcribed RNA genome into the host genome. A stable lentiviral vector (LVV) packaging cell line, GPRTG, was recently developed by sequentially integrating essential viral structural and replication genes into HEK293T/17 cells [1]. This LVV packaging cell line contains all necessary components for LVV production, except the gene of interest.

To generate LVV producer cell lines, the gene of interest (GOI) must be stably integrated into the genome, traditionally through random insertion of plasmids or concatemeric arrays [1]. This process often results in a polyclonal, heterogeneous cell populations with diverse genetic characteristics [2], as integration into transcriptionally inactive sites may affect gene silencing/activation of endogenous genes. Consequently, the polyclonal mixture will contain cells with low and high GOI expression entailing intensified screening for suitable production clones are the encountered drawbacks [3]. In addition, high pool‐to‐pool variability resulting from random transgene integration is leading to variance during cell cultivation.

To overcome these challenges and to control GOI integration, more directed integration approaches have been developed including recombinase‐based, CRISPR/Cas9‐mediated, and transposase‐mediated systems, which all enable site‐specific integration [4, 5, 6]. Transposases, in particular, have gained prominence in genetic engineering, with key systems including Sleeping Beauty, piggyBac, and Tol2 transposases [7, 8]. Transposition occurs through a mechanism known as “cut and paste”, where the transposase excises the GOI cassette flanked by inverted terminal repeats (ITR) from a transfected plasmid and integrates it semi‐targeted into the genome of the target cell line [9]. Here, the piggyBac transposase enables seamless integration without leaving residual sequences at the insertion site, distinguishing it as a unique system among the other systems [7]. In addition, it integrates DNA preferentially near transcriptional start sites in genomic regions [10, 11], which is advantageous for GOI expression, but also carries the risk of disrupting the transcriptional activity of essential endogenous genes. In 2011, Yusa et al. identified a hyperactive variant of the piggyBac transposase, enhancing the potential for gene delivery and the development of stable production cell lines [12]. This variant harbor specific amino acid substitutions that significantly increase transposition activity, resulting in higher rates of transgene integration. The enhanced catalytic efficiency facilitates more robust and stable genomic insertion, thereby improving the overall effectiveness of gene delivery, particularly in cell types with low transfection or integration efficiency.

In the past, the LVV producer GPRTG cell lines for the therapeutical approaches for Wiskott–Aldrich syndrome (WAS) and X‐linked agammaglobulinemia (XLA) have been generated using concatemeric‐array transfection [1, 13]. However, this approach requires prolonged selection phases, is susceptible to mutations due to the nature of the generation process, demands large amounts of purified DNA and is time consuming. This raises the question if alternative approaches as transposition can improve the efficiency, stability, and scalability of LVV producer cell line generation.

This study investigates two distinct approaches for the generation of stable cell lines—concatemeric integration and transposase‐mediated integration—with a focus on comparing their efficiencies in antibiotic selection recovery, cellular growth kinetics during LVV production, and their influence on final LVV titers. By delineating the relative performance of these strategies, the study aims to inform the optimization of stable producer cell line development for enhanced LVV manufacturing efficiency.

## 2 Material and Methods

### 2.1 Cell Culture

The generation of GPRTG cells utilized in this study has been previously described [9]. These cells were cultured in HyCell TransFx‐H medium (Cytiva, Marlborough, MA, USA) supplemented with 6 mM GlutaMAX (Thermo Fisher Scientific, Schwerte, Germany), 1.9 g/L Cell Boost 5 stock solution (Cytiva, Marlborough, MA, USA), 0.1% (w/v) Pluronic F‐68 non‐ionic surfactant (Thermo Fisher Scientific), and 0.01% (v/v) anti‐clumping agent (Thermo Fisher Scientific). Cultivation was carried out in a shaking incubator at 37°C with 120 rpm (25 mm orbit), under conditions of 5% CO_2_ and 85% humidity. GPRTG cells were passaged every 3–4 days. Viability and viable cell density were assessed using the NucleoCounter NC‐200 with Via1‐Cassettes (ChemoMetec A/S, Allerod, Denmark), following the manufacturer's instructions and employing the viability and cell count imaging method (NucleoView software version 1.3.0.0).

### 2.2 Plasmid DNA Construction

#### 2.2.1 Concatemeric Array Construction

The concatemeric arrays for the generation of stable producer cell lines were generated as previously described by Throm et al. and Klimpel et al. Either XLA or a WAS‐T2A‐GFP construct was cloned into a tetracycline‐regulated pTL20 vector backbone. The pTL20 and pPGK‐ble resistance vector were amplified through bacterial transformation, followed by Giga preparation for plasmid purification, as per the manufacturer's protocol (QIAGEN Inc.). Both plasmids were linearized using PfIMI and SfiI (New England Biolabs GmbH). Following digestion, the fragments were ligated in a molecular ratio of 25:1 using T4 DNA ligase (New England Biolabs GmbH) overnight. The constructs were purified using Genomic‐tip DNA extraction kit (QIAGEN inc.) and resuspended in TAE buffer.

#### 2.2.2 Transposon Plasmid Construction

**FIGURE 1:**
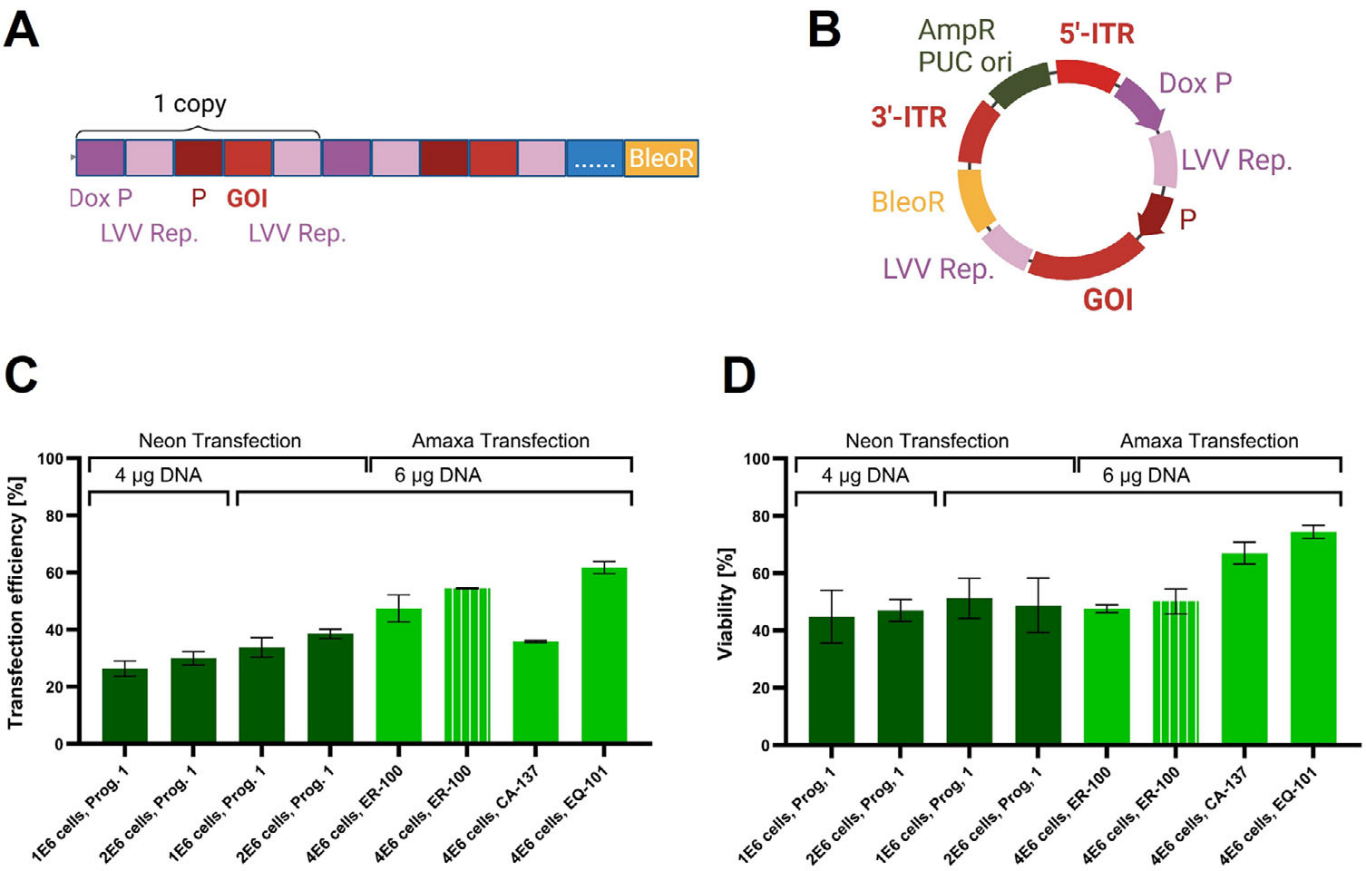
**DNA constructs and transfection optimization**. Schematic depiction of a concatemeric‐array (A) and a transposon vector (B). Dox P = doxycycline regulated promotor, LVV Rep. = Sequences necessary for LVV packaging, P = Promotor, GOI = Gene of interest, […. .] = placeholder for multiple copies of GOI, BleR = bleomycin resistance marker, AmpR = ampicillin resistance marker, ITR = inverted terminal repeat. Transfection optimization overview of different protocols and the effects on transfection efficiency (C) and cell viability (D) (fully colored bars = shaken cultivation, striped bar = static cultivation).

A schematic depiction of the used transposon vectors is shown in Figure 1A.

To construct the final XLA transposon vector AuGTGd23‐04_5426Fw (pTransposon), the BstBI, SspI, and HpaI fragment of 5426fw plasmid (insert fragment) carrying the transgene‐lentiviral expression cassette was cloned into BstBI and HpaI digested vector backbone AuGTGd23‐03. Vector backbone AuGTGd23‐03 containing the piggyBac ITRs was synthesized by Genescript. The final plasmid AuGTGd23‐04_5426Fw with transposon (Figure 1) was analyzed via control restriction digestion and checked with gel electrophoresis and isolated using QIAquick Gel Extraction Kit (QIAGEN inc.). After confirmation, the transposon vector was transformed into chemical competent *Escherichia coli* cells in LB broth and prepared using QIAGEN Plasmid Midi Kit (QIAGEN inc.).

To construct the final WAS‐GFP transposon vector, the XLA transgene cassette, including the XLA promoter and XLA sequence, was replaced with the MND promoter and WAS‐GFP sequence. A schematic representation of the WAS‐GFP transposon vectors used is shown in Figure 1B. The final construct was analyzed via control restriction digestion, verified with gel electrophoresis, isolated using the QIAquick Gel Extraction Kit, transformed into NEB Stable Competent *E. coli* Cells (New England Biolabs GmbH), and plasmids were prepared using the QIAGEN Plasmid Midi Kit (QIAGEN inc.).

#### 2.2.3 Linearized Plasmid Generation

To generate linearized WAS‐GFP and XLA vectors, the Transposon plasmids were digested using the restriction enzymes PmeI and NsiI (New England Biolabs GmbH). Following digestion, the DNA fragments were separated using gel electrophoresis and isolated using QIAquick Gel Extraction Kit (QIAGEN inc.) and the concentration was measured using NanoDrop (Thermo Fisher Scientific).

### 2.3 DNA Vector Transfection

#### 2.3.1 Electroporation

For transfection of GPRTG cells in 24‐well plates, two different systems were used: the Neon transfection device (Thermo Fisher Scientific) with 10 and 100 µL tips and the Amaxa 4D Nucleofector.Various Neon transfection settings were optimized, including different cell seedings (1, 2, and 4 * 10^6^ cells/transfection) and total DNA inputs (1, 2, 4, and 6 µg) of a GFP‐expressing plasmid. For the Amaxa 4D Nucleofector, the programs ER‐100, FF‐132, CM‐130, CA‐137, and EG‐101 were tested, along with different cell amounts (2 and 4*10^6^ cells/transfection) and total DNA inputs (4 and 6 µg). Additionally, both static and shaken cultivation methods were evaluated post‐transfection. Table 1 summarizes the transfection conditions tested. As a negative control, cells were used either with or without an electric pulse but without DNA transfection.

**TABLE 1:** Overview of tested transfection conditions.

Well‐format	Device	Cells/well	DNA input/well	Program	Note
24	Neon	1*10^6^	4 µg	1	n.a.
24	Neon	2*10^6^	4 µg	1	n.a.
24	Neon	1*10^6^	6 µg	1	n.a.
24	Neon	2*10^6^	6 µg	1	n.a.
24	Amaxa	4*10^6^	6 µg	ER‐100	Cultivated shaken post transfection
24	Amaxa	4*10^6^	6 µg	ER‐100	Cultivated static post transfection
24	Amaxa	4*10^6^	6 µg	CA‐137	n.a.
24	Amaxa	4*10^6^	6 µg	EQ‐101	n.a.

#### 2.3.2 Lipofection

For the generation of stable producer cell lines using concatemeric arrays the GPRTG cell line was seeded 24 h prior transfection with 2.21*10^6^ cells in a 60 mm dish using D10 media with a final volume of 5 mL. On day of transfection the media was replaced with Opti‐MEM to a final volume of 2.5 mL. Transfection was performed using Lipofectamine 3000/2000 (Thermo Fisher Scientific) according to the manufacturer's manual, with a P3000/P2000 to DNA ratio of 1:1, ranging from 11.1 µg DNA to 62.5 µg DNA.

### 2.4 Selection Process

For concatemer‐based pools, Zeocin selection began 72 h after transfection at an initial concentration of 50 µg/mL. Once cell growth resumed, the Zeocin concentration was lowered to 20 µg/mL, and the cells were subsequently cultured under serum‐free conditions in shake flasks.

For transposase‐based pools, selection also started 72 h post transfection, using two fixed Zeocin concentrations (20 and 40 µg/mL). These concentrations were maintained throughout the entire selection period.

### 2.5 LVV Production

The selected cell pools were inoculated into 125 mL shaking flasks at an initial viable cell density of 1.5*10^6^ cells/mL. The pools were cultured in batch mode for 72 h in doxycycline‐free HyCell TransFx‐H (Cytiva) cultivation medium. Starting 72 h post‐induction, a daily media exchange and sampling were carried out. For this, the cell culture was centrifuged at 800 − *g* for 2 min, after which the LVV material in the supernatant was aliquoted and stored at −80°C. The cell pellet was then resuspended in fresh cultivation medium, marking one harvest. Sampling continued until six harvests were completed.

### 2.6 Infectious Titer Determination

#### 2.6.1 Infectivity Assay by FACS for WAS‐GFP Constructs

Functional LVVs in the cell culture supernatant were quantified using a transduction assay with HEK293T/17 cells. Adherent HKE293T/17 cells were cultured in DMEM medium (Thermo Fisher Scientific) supplemented with 10% fetal bovine serum (FBS) (Thermo Fisher Scientific) and 1% Penicillin‐Streptomycin (Thermo Fisher Scientific) at 37°C and 5% CO_2_. The infectivity assay was performed in 24‐well plate or 96‐well plate format. For the 24‐well format, the cells were seeded in 800 µL/well DMEM cultivation media supplemented with 125 µg/mL polybrene (Merck) (DMEM transduction media) at a VCC of 1*10^5^ cells/well in. LVV containing supernatant samples were thawed quickly and serial diluted in DMEM transduction media. The 4‐point dilution series included dilution factors of 5, 25, 125, and 625. The LV tracking control with a known concentration was diluted 25.000‐fold. DMEM transduction media serve as negative control. Two hundred microliters of diluted LVV harvests, tracking control or negative control were added to the 800 µL seeded cells, mixed by pipetting up and down and incubated 37°C and 5% CO_2_ for 4 days.

For the 96‐well format, the HEK293T/17 cells were seeded in in 80 µL/well DMEM transduction media at a VCC of 2*10^4^ cells/well. LVV harvest was diluted with a 4‐point serial dilution including dilution factors of 50, 250, 1250 and 6250. The tracking control was 50.000‐fold diluted. Twenty microliters of diluted LVV harvests, tracking control and negative control were added to 80 µL seeded cells, mixed and incubated at 37°C and 5% CO_2_ for 4 days.

Ninety‐six hours post transduction, the cells were washed with PBS, trypsinized with TrypLE express (Thermo Fisher Scientific), incubated at 37°C for 3 min and mixed with DMEM cultivation media to stop the enzymatic reaction. Cells were fixed using the BD Biosciences Fixation and Permeabilization Kit, following the manufacturer's instructions, or with 4% paraformaldehyde (PFA), followed by two washes with PBS. Subsequently, cells were resuspended in 100 µL of MACS Running Buffer (Miltenyi Biotec) and analyzed for GFP‐positive populations using the CytoFLEX flow cytometry system (Beckman Coulter Life Sciences).

### 2.7 Infectivity Assay by Droplet Digital PCR for XLA Constructs

Functional LVVs in the cell culture supernatant were quantified using a transduction assay with adherent HEK293T/17 cells. The cells were transduced with LVV harvests to determine functional LVV titers. Specifically, 3*10⁶ cells were resuspended in 12 µL polybrene (10 mg/mL, Millipore) to a final volume of 12 mL using DMEM cultivation media. A multichannel pipette was employed to dispense 80 µL of the cell suspension into each well of a 96‐well plate.

#### 2.7.1 LVV Harvest Dilution and Transduction

LVV harvests were serially diluted 5‐, 50‐, and 500‐fold, while the lentiviral (LV) tracking control was diluted 5000‐fold. Subsequently, 20 µL of each diluted harvest or tracking control was combined with 80 µL of cell suspension and incubated for 4 days at 37°C with 5% CO_2_.

#### 2.7.2 Cell Lysis and DNA Extraction

Ninety‐six hours post‐transduction, cells were lysed using 75 µL of the Direct PCR DNA extraction system (VWR) according to the manufacturer's instructions. The extracted samples were then diluted 1:20 in nuclease‐free water.

#### 2.7.3 Droplet Digital PCR (ddPCR) Setup

For ddPCR, 4 µL of each diluted sample was mixed with 18 µL of ddPCR master mix. Each 24 µL reaction mix contained 1× ddPCR Supermix for Probes (no dUTPs) (Bio‐Rad), 1100 nM forward primer, 1100 nM reverse primer, and 611 nM probe (Bio‐Rad). The reaction mix was loaded into the QX100 droplet generator (Bio‐Rad) to generate droplets, following the manufacturer's instructions.

#### 2.7.4 PCR Conditions

After droplet generation, PCR was conducted according to the settings outlined in Table 2.

**TABLE 2:** Overview of thermocycler settings for PCR.

Step	Temperature	Time (min.:sec.)	Cycles
Enzyme activation	95°C	10:00	1×
Denaturation	94°C	0:30	39×
Annealing/Extension	57°C	1:00	
Enzyme deactivation	98°C	10:00	1×
Hold	4°C	∞	∞

**TABLE 3:** Overview of PCR primer used for amplification.

Name	Sequence	Modification
WPRE‐FW	5'‐ GCT ATG TGG ATA CGC TGC TTT A ‐3'	n.a.
WPRE‐RV	5'‐ AGA GAC AGC AAC CAG GAT TTA TAC ‐3'	n.a.
WPRE‐PB	5'‐ TC ATG CTA TTG CTT CCC GTA TGG CT ‐3'	FAM /ZEN ‐Iowa Black FQ
Albumin‐FW	5'‐ GCT GCT ATC TCT TGT GGG CTG T ‐3'	n.a.
Albumin‐RV	5'‐ ACT CAT GGG AGC TGC TGG TTC ‐3'	n.a.
Albumin‐PB	5'‐ CCT GTC ATG CCC ACA CAA ATC TCT CC ‐3'	HEX/ZEN ‐Iowa Black FQ

After PCR amplification, positive and negative droplets were quantified using the QX200 droplet reader (Bio‐Rad) and QuantaSoft software (Bio‐Rad), following the manufacturer's instructions.

The following primer‐probe set was used for PCR amplification (Table 3):

## 3 Results

### 3.1 Transfection Optimization for Transposon Based GOI Integration

Previously, the GPRTG packaging cell line was developed through sequential stable genomic integration of viral genes into an adherent HEK293T/17 cell line using gamma retroviral vectors [13]. Subsequently, stable producer cell lines were established by lipid‐mediated transfection of the packaging cell line using concatemeric arrays containing the GOI. These concatemeric arrays consist of multiple copies of a linearized DNA cassette containing the gene of interest flanked by sequences necessary for viral packaging ligated to a single copy of a linearized antibiotic resistance vector at a molecular ratio of 25:1 [13], facilitating high‐copy‐number genomic integration (Figure 1A). As the generation of those arrays is laborious and renders the gene sequence susceptible to mutations, employing a transposase system could offer a valuable alternative method. To enable semi‐targeted transposase‐mediated genome integration, a circular transposon vector was generated. This vector builds on previous vectors designed for concatemeric arrays and includes ITR for PiggyBac transposase to facilitate the integration process (Figure 1B).

For the establishment of efficient transfection of the new transposon vector into packaging GPRTG cells, electroporation was chosen to enable gene integration into the genome, since previous experiments displayed difficulties using other transfection methods. As stable GOI integration a process that heavily relies on how effectively the cells take up the vector during transfection, electroporation was optimized by a systematic evaluation of various devices, electroporation parameters, DNA concentrations, and cell seeding densities. Finally, transfection efficiency was measured based on the expression of the GFP transgene of a model vector.

The Neon device achieved the highest transfection efficiency using 2*10⁶ cells, 6 µg of total DNA, and program 1 (Figure 1C). Moreover, cell viability remained comparable to other tested conditions, around 50% (Figure 1D). In contrast the Amaxa device, paired with a cell seeding density of 4*10⁶ cells per well and 6 µg of total DNA, demonstrated the highest transfection efficiencies, ranging from 37.8% to 61.7%. Although cell viability was generally similar across conditions tested, the Amaxa EQ‐101 program stood out, achieving the highest transfection efficiency (61.7%) while maintaining robust cell viability (74.3%) (Figure 1C,D). Therefore, this setting was selected for all further experiments.

### 3.2 Stable Cell Line Generation Using Concatemeric Arrays and Transposase System

**FIGURE 2:**
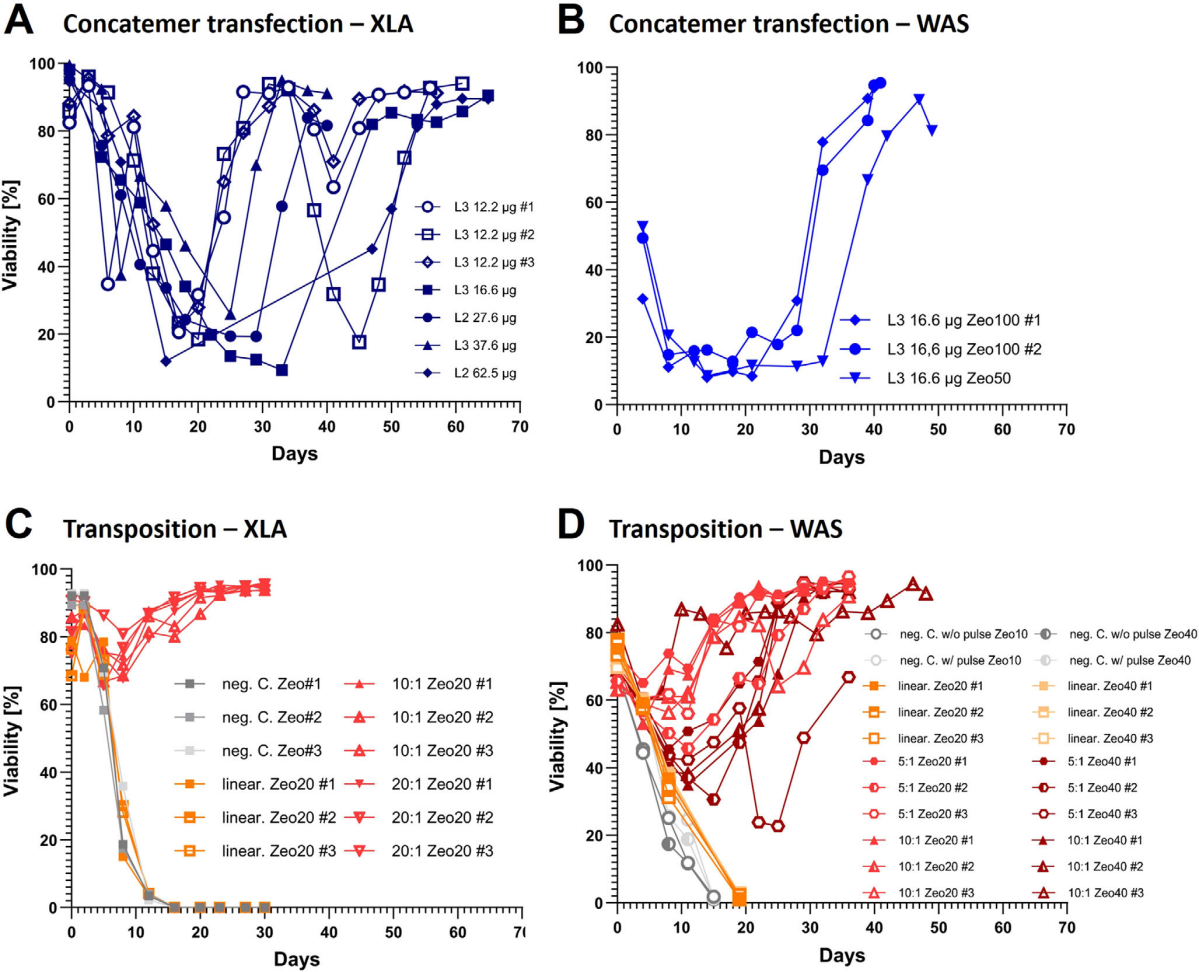
**Stable producer cell pool generation process**. Selection process overview for concatemeric arrays with XLA GOI (A) and WAS‐GFP GOI (B) and transposase‐based pools for XLA (C) and WAS‐GFP (D). L3 = Lipofectamine 3000, L2 = Lipofectamine 2000, Zeo20/40/50/100 = Zeocin concentration (µg/ml), 12.2–62.5 µg = total DNA used, # = replicate, neg. C. = negative Control, linear. = linearized transposon plasmid, 5:1/10:1/20:1 = ratio transposon vector to transposase vector.

To establish stable production cell lines, it is essential to select for stably integrated gene copies. Following transfection of concatemeric arrays or transposon and transposase vectors, cell pools were treated with zeocin to enrich for stable integrants, ultimately resulting in polyclonal stable cell populations. Investigating differences in the duration of the selection process, we compared two transgenes, XLA and WAS‐GFP, using both concatemeric arrays and transposase‐based systems. Concatemeric array transfection and selection exhibited the longest and more varied recovery times to reach over 95% viability, ranging between 30 and 65 days, with an average of approximately 50 days (Figure 2A,B). A second crisis and reduced cell viability was observed after removal of FBS on Day 35 for pools transfected with 12.2 µg DNA of XLA concatemeric arrays. Although this delayed cell recovery to up to 65 days, pools transfected with higher DNA amounts of >16.6 µg and Lipofectamine 3000 (L3) only encountered a single crisis phase and demonstrated faster recovery. Here, the shortest recovery time was observed at 33 days (clone L3 37.6 µg) and most pools required around 40 days for recovery. In all XLA concatemeric pools, the initial viability crisis deep ranging from 10% to 22% viability, while a second crisis appeared in pools transfected with 12.2 µg of DNA resulting in greater variability, ranging from 18% to 71%. Possibly, the integrated DNA levels are too low to prevent a second crisis—an effect that may be avoided by using higher amounts of DNA. As lower DNA amounts seemed to increase recovery times and are more cost‐efficient, concatemeric arrays carrying the WAS‐GFP transgene were transfected with intermediate DNA amount of 16.6 µg DNA, which led to only a single crisis phase and no effect due to FBS removal (Figure 2B). Recovery times for these stable pools ranged from 40 to 46 days for full recovery.

In contrast to concatemeric arrays, transposase‐system transfected cell pools using above established conditions using only 6 µg DNA, demonstrated faster recovery times, a less deep viability crisis and more homogenous recovery dynamics. For the XLA transgene, stable pools generated using the transposase system recovered particularly quickly, ranging from Day 16 to 25 to exceed viability of 95% (Figure 2C). Varying the ratio of circular transposon to transposes plasmids between 20:1 and 10:1 did not induce major differences. Only linearized plasmids and negative controls failed to survive the selection phase, while all other clones recovered after a viability crisis, which was much milder than in concatemer based cell pools, with viabilities not decreasing below 66%.

Transposase‐based systems carrying the WAS‐GFP transgene also outperformed concatemeric arrays in recovery speed. Cell pools transfected with linearized vectors did not survive the selection period, while all other cell pools recovered within 20–30 days post‐transfection, except pool 5:1 Zeo40 #3 and 10:1 Zeo40 #3 (Figure 2D). Viability crisis was again far less deep as in concatemer based cell pools; however, raising the Zeocin concentration from 20 to 40 µg resulted in a more pronounced crisis, while increasing the transposon amounts (5:1 ratio) did not significantly impact the depth of the viability crisis.

Overall, concatemeric arrays exhibited longer and more variable recovery times, particularly under conditions with lower DNA inputs. In contrast, transposase‐based systems consistently achieved faster and more reliable recoveries with mild crisis for both XLA and WAS‐GFP transgenes, highlighting their efficiency in stable cell line generation.

### 3.3 Influence of Pool Generation Method and DNA Construct on LVV Producer Pool Growth Performance

To evaluate whether the method used for generating stable LVV producer cell pools affected growth parameters, all generated stable cell pools were seeded and induced to produce LVVs by removing doxycycline.

**FIGURE 3:**
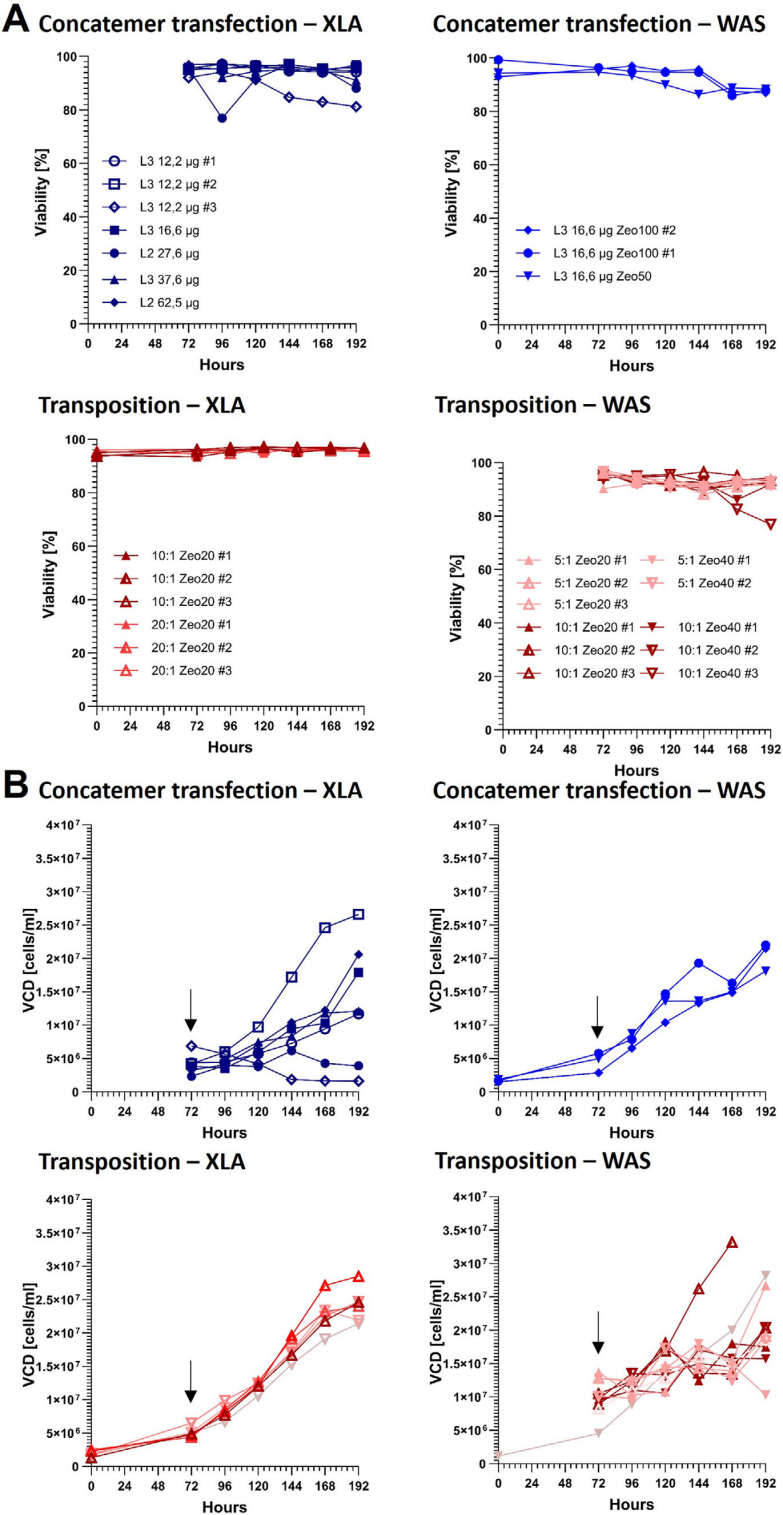
**Stable producer cell pool LVV production process**. Viability during LVV production process for concatemeric arrays and transposase‐based pools (A) and viable cell density (VCD, B). Black arrows indicate the starting point of daily media exchange and harvest collection. L3 = Lipofectamine 3000, L2 = Lipofectamine 2000, Zeo20/40/50/100 = Zeocin concentration (µg/ml), 12.2–62.5 µg = total DNA used, # = replicate, neg. C. = negative Control, linear. = linearized transposon plasmid, 5:1/10:1/20:1 = ratio transposon vector to transposase vector.

Throughout the production period, cell viability across most pools, whether generated using concatemeric or transposase arrays, remained consistently around 90% until 168 h post induction (Figure 3A). A slight drop in viability of 20% was only observed for a few individual cell pools, including XLA concatemeric array‐based cell pool at 144 h post induction (L3 12,2 µg #3) and WAS transposase‐based cell pool at 168 h for WAS‐GFP (10:1 Zeo40 #3, Figure 3A).

Although the average viability was relatively similar, the viable cell density among individual concatemeric‐based and transposase‐based pools was more variable. Although XLA concatemeric array‐based pools showed high VCD variability ranging from 1.63*10⁶ to 2.66*10⁷ cells/mL (Figure 3B), WAS‐GFP pools generated with concatemeric arrays displayed a more consistent VCD, ranging from 1.81*10⁷ to 2.20*10⁷ cells/mL (Figure 3B). In contrast, XLA pools generated using transposase exhibited less variability, with cell densities ranging from 2.14*10⁷ to 2.85*10⁷ cells/mL, whereas WAS‐GFP transposase‐based pools displayed greater variation, with densities ranging from 1.03*10⁷ to 3.32*10⁷ cells/mL (Figure 3B).

In general, the choice of transfection method, whether electroporation and transposase‐based pool generation or lipofection and concatemeric‐array‐based pool generation, had no impact on pool growth‐stability during LVV production, as transfection‐associated stress occurs only for a limited time‐period.

### 3.4 Functional Titer Analysis and Correlation Analysis of LVV Producer Pools

To evaluate the impact of the method used for generating stable LVV producer cell pools on their performance, specifically in terms of functional LVV titers and the likelihood of obtaining high‐producing cell pools, comprehensive titer analyses were conducted for all cultivated producer pools. Cell culture supernatants were collected daily starting from 72 to 195 h post‐induction, infectivity assays performed, and LVV titers determined using flow cytometry for WAS‐GFP and digital droplet PCR (ddPCR) for XLA transgene expression.

**FIGURE 4:**
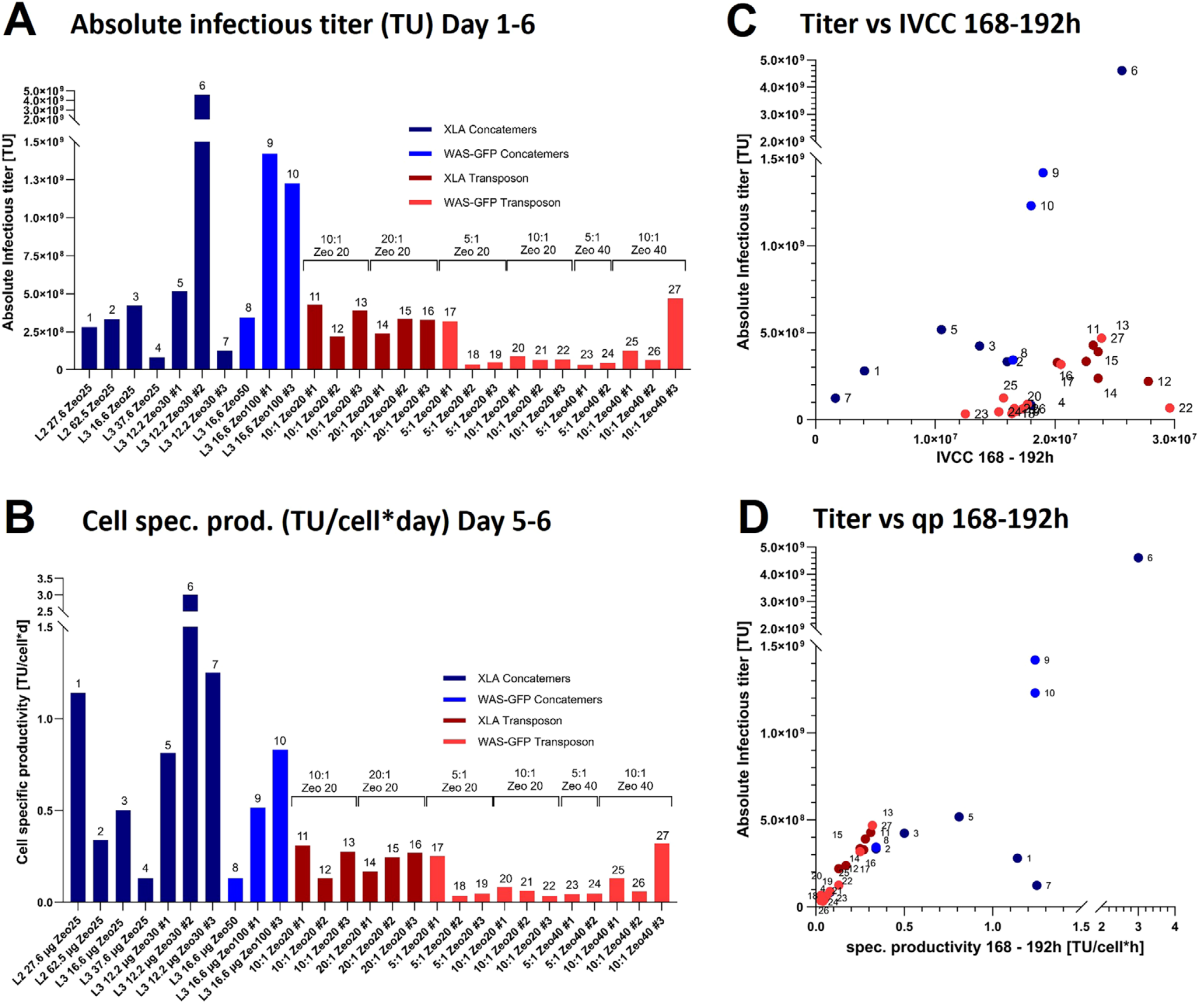
**Infectious titer analysis and correlation analysis**. Infectious titer (A) and cell specific productivity (B) for concatemeric arrays and transposase‐based producer pools. Comparisons of absolute infectious titer with IVCC (C), and cell specific productivity (D) between 168–192 h. L3 = Lipofectamine 3000, L2 = Lipofectamine 2000, Zeo20/40/50/100 = Zeocin concentration (µg/ml), 12.2–62.5 µg = total DNA used, # = replicate, neg. C. = negative Control, linear. = linearized transposon plasmid, 5:1/10:1/20:1 = ratio transposon vector to transposase vector.

For infectious titer comparison, the supernatant of each harvest timepoint was pooled and the absolute infectious titer (from Day 1 to Day 6) determined. Concatemeric‐based producer pools displayed notable variability in infectious titers, with the majority yielding low to medium titers, while a few pools produced very high titers (Figure 4A). The highest infectious titer of 4.6*10⁹ TU was produced by XLA concatemer‐based pool (L3 12.2 Zeo30 #2), followed by two concatemer‐based WAS‐GFP pools with 1.2*10⁹ and 1.4*10⁹ TU (L3 16.6 Zeo100 #1 and L3 16.6 Zeo100 #3). The infectious titers of the remaining pools were lower, ranging between 8.2*10⁷ and 5.2*10⁸ TU. In contrast, transposase‐based pools exhibited more consistent titers, with XLA pools ranging from 2.2*10⁸ to 4.3*10⁸ TU, and WAS‐GFP pools from 3.2*10⁷ to 4.7*10⁸ TU (Figure 4A). However, very high‐producing pools as generated for concatemeric‐based GOI integration were not achieved for neither transgene, and WAS‐GFP pools generally exhibited a lower titer than XLA expressing pools.

To better understand the production efficiency of individual cells, cell‐specific LVV productivity was analyzed. Concatemeric‐based pools generally exhibited higher cell‐specific productivities compared to transposase‐based pools; however, also showing a wider range of variability in cell‐specific productivity (Figure 4B). Among them, the concatemeric‐XLA pools demonstrated the highest productivity, with values ranging from 0.3 to 3 TU/cell*day, followed by the concatemeric‐based WAS‐GFP pools, which ranged from 0.1 to 0.8 TU/cell*day. In contrast, transposase‐based pools displayed more consistent, yet lower, cell‐specific productivities, with values ranging from 0.1 to 0.3 TU/cell*day for XLA and 0.03 to 0.3 TU/cell*day for WAS‐GFP (Figure 4B).

To identify parameters that may distinguish high‐producing cell pools during production, we correlated absolute infectious titers of pooled supernatants from 72 to 192 h post induction to various production parameters. Factors such as viability, time to reach >90% viability during selection, VCD during production, total DNA input, selection marker amount, and specific growth rate from 168 to 192 h showed no correlation with infectious titer levels (Figure S1). However, a clear trend was observed for high‐producing cell pools consistently exhibiting a high integral of viable cell concentration (IVCC) from 168 to 192 h post‐induction (Figure 4C), suggesting a link between increased titer and fast cell growth. In addition, the high producing pools exhibited elevated cell specific productivities, underscoring a link between overall productivity and the efficiency of individual cells within these pools (Figure 4D).

In summary, the results indicated that robust cell growth and high cell‐specific productivity can be key drivers of overall productivity, with growth characteristics serving as reliable indicators for high‐producing pools. However, concatemeric‐arrays exhibited more diverse but the highest LVV titers, whereas transposase‐based pools showed more consistent lower LVV titers.

## 4 Discussion

Biopharmaceutical companies that use stable LVV producer cell lines for gene therapy applications are in need for an optimized production platform, streamlining the generation process for production cell lines, reducing costs, improving consistency, and minimizing batch‐to‐batch variability [14]. The suspension‐adapted GPRTG packaging cell line is offering a solution to challenges associated with transient LVV production [13]. However, the establishment of stable producer cell lines based on GPRTG packaging cell line currently relies on concatemeric array integration, which is susceptible to mutations due to the nature of the generation process and demands large amounts of purified plasmid DNA during generation process [1].

In order to access improvement of this process, we compared concatemeric array integration to transposase‐based genome integration using hyperactive piggyBac transposase. By analyzing key aspects as the selection process, growth characteristics, and resulting LVV titer for two different transgenes, we aimed to identify suitability of these methods for producing high‐yield and consistent LVV producer cell lines.

The efficiency of plasmid transfection is crucial for the success of transposase‐mediated integration. The achieved high transfection efficiencies for electroporation suggested that the piggyBac transposase system in combination with optimized electroporation could be a viable alternative for generation of stable producer cell lines. The subsequently performed selection process for stable producer pools is time‐consuming and shorter timelines highly desirable. Concatemer transfected cells required long and variable recovery times. In contrast, the cellular crisis during selection was less pronounced when using the transposase‐based approach, additionally exhibiting more consistent and accelerated recovery times reducing hands‐on labor associated with stable cell line generation. Additionally, the transposase‐based approach obviates the need for fetal bovine serum (FBS), thereby addressing associated safety concerns [15], regulatory and ethical issues [16], supply chain inconsistencies [17], and high costs [18]. Combined with the substantially lower DNA requirements, this strategy offers a significant reduction in overall production expenses. In our hands, linearized plasmids for both XLA and WAS‐GFP failed to yield stable cell lines under selection. This may be due to the increased susceptibility of linear DNA to intracellular nuclease degradation, which can reduce the likelihood of successful genomic integration [19]. Additionally, linear DNA may be more prone to fragmentation during electroporation, further compromising its stability. The intracellular trafficking of linear DNA is typically less efficient and can be strongly influenced by the chosen delivery method. For example, electroporation, while highly effective for some applications, may cause additional strand breaks in linear DNA, further compromising its stability and integration potential [19]. In contrast, lipofection has been shown to be less damaging to linear constructs and may enhance nuclear delivery. For example, Wang et al. demonstrated stable genomic integration of in vitro–ligated double‐stranded DNA fragments into HEK293T cells using lipofection, highlighting that delivery method can be a critical determinant of success [20]. These findings suggest that optimization of the transfection strategy is critical when working with linear DNA.

During the LVV production process, robust growth and sustained high viability allow for extended LVV production maximizing overall yield. For both approaches, cell viability remained consistently high throughout the LVV production process, suggesting the potential for extending production beyond 7 days. This surprising extended production time was first described in 2023 by Klimpel et al. [2]), and contrasts with other published LVV production cell lines previously used for large‐scale studies and reporting LVV production durations of only 4–7 days using [22, 23, 24]. However, viable cell density exhibited greater variability depending on the gene of interest and the transfection method in small scale model, and may therefore be a valuable target for further optimization to enhance reproducibility and improve the efficiency of LVV production.

Following the LVV production process, the infectious titer of each transfection approach was assessed and revealed distinct production profiles. Concatemeric‐based pools exhibited substantial variability, with a small subset of cell pools producing exceptionally high titers of 4.6*10^9^ TU/mL, aligning with previous studies demonstrating that concatemeric arrays can support high LVV titers [22]. In contrast, transposase‐based pools generated more consistent but generally lower infectious titers reaching 4.7*10^8^ TU/mL. This suggests that transposase‐mediated integration leads to more uniform, yet less extreme productivity levels compared to concatemeric integration. However, comparing the transposase‐based pools to other inducible lentiviral producer cell lines, the performance is competitive with established systems such as ProSavin, which achieves titers of 3*10^5^ TU/mL, or the cell line reported by Milani et al., producing 4.4*10^6^ TU/mL [24, 25]. Although the stable producer cell line used for ProSavin production is an adherent cell line, developed via lipofectamine‐mediated transfection of a linearized GOI containing plasmid, a more comparable system is the HEK293SF‐based suspension cell line developed by Broussau et al., which yields an LVV titer of approximately 3*10^6^ TU/mL [23] utilizing a Tet‐On system to activate LVV production. An additional suspension‐based producer cell line was reported by Tridgett et al., where piggyBac transposase system was used for cell generation and high LVV titers of 2.3*10^8^ TU/mL obtained. However, their analysis was conducted after single‐cell cloning, where high producing clones were be selected out of a producer pool [26]. The consistently lower titers observed in WAS‐GFP pools compared to XLA pools may be attributed to several transgene‐specific factors. One key aspect is the difference in transgene length: WAS‐GFP is approximately 300 base pairs longer than XLA. Previous studies have demonstrated that increasing the size of the lentiviral genome negatively affects packaging efficiency and viral yield. For instance, Han et al. reported that a 1 kb increase in vector length can reduce titer by up to 50%, due to impaired reverse transcription and reduced virion stability [23]. Additionally, the bicistronic architecture of WAS‐GFP, which includes a T2A peptide, may introduce RNA secondary structures that interfere with transcription and reverse transcription. The translation of two proteins from a single transcript also imposes a higher metabolic burden on producer cells, potentially limiting overall productivity [27]. These combined factors likely contribute to the reduced titers observed for WAS‐GFP pools

Although transgene copy number is a known determinant of expression and productivity, we deliberately refrained from quantifying copy number in bulk populations due to the inherent heterogeneity of integration events. In polyclonal pools, copy number measurements represent only averaged values and may obscure biologically relevant differences between subpopulations [28]. Moreover, techniques such as ddPCR and Southern blotting, while powerful in clonal contexts, offer limited resolution in mixed populations and are prone to underestimating complex integration patterns [4]. Future studies incorporating clonal isolation and targeted copy number analysis will be essential to dissect the relationship between integration architecture and productivity. This will allow us to build on the current findings and further optimize transposase‐based integration strategies.

To identify key determinants of overall productivity and establish indicators for the rapid selection of promising pools for single‐cell cloning, the correlation analysis between absolute infectious titers and various process parameters and cell characteristics. The analysis revealed that high‐producing pools consistently exhibited greater IVCC, VCD, and cell‐specific productivity, suggesting that elevated titers result from both robust cell growth and a higher proportion of productive cells and are key drivers of overall productivity. Furthermore, these parameters could serve as valuable selection criteria for identifying high‐producing pools early in the process of generating stable LVV producer cell lines and optimize production efficiency.

The generated data both underscore the potential of transposase‐based approaches to be successfully used for the generation of LVV producing cells, and also underscore key trade‐offs between the two integration methods.

The concatemeric‐based integration can result in some cases in cell pools exhibiting exceptionally high titers and cell‐specific productivity; however, it also introduces significant variability during the cell pool generation process. In contrast, transposase‐based cell pools exhibit low variability in selection recovery and growth behavior, but fail to achieve high LVV yields on pool level. In addition, the transposase‐based approach needs far lower DNA inputs than concatemeric‐array transfections, which makes it cost effective and also suggests potential for further optimization of the transposase system. Transposase‐based systems streamline clinical development by supporting stable polyclonal pools for early‐phase manufacturing and efficient monoclonal line generation for late‐phase production. Their consistent integration mechanism improves comparability and limits variability in transgene structure and expression, whereas concatemeric integration often produces heterogeneous patterns that hinder process consistency and regulatory alignment. In combination with single‐cell cloning to identify high‐performing clones, the transposase approach appears highly competitive with concatemeric‐based applications.

## Author Contributions

K.O., H.L., and P.G. supervised the project. J.R., J. H., V.P., and M.K. planned and conducted experiments. H.D. assisted J.R. with the transposon vector cloning. J.R. analyzed and evaluated data and wrote the initial manuscript. All authors read the final manuscript and provided critical feedback.

## Ethics Statement

The experiments of this study were conducted without animal material and therefore not critical in regard of ethics.

## Conflicts of Interest

The authors declare that they have no known competing financial and non‐financial interests or personal relationships that could have appeared to influence the work reported in this paper.

## Supporting information




**Supporting Information file 1**: biot70135‐sup‐0001‐FigureS1.docx

## Data Availability

The datasets generated and analyzed during the current study are not publicly available due company internal issues, but are available from the corresponding author on reasonable request after clarifying permission with Dr. Holger Laux and CSL Behring innovation GmbH.

## Appendix A

### A.1

The data in this section contains additional correlation analysis between absolute infectious titer with IVCC for the whole LVV production period, specific growth rate μ between 168 and 192 h post induction and whole LVV production period, viability 192 h post induction, amount of selection marker, and total DNA transfected.
