# Supplementary material for: Optimizing Lentiviral Vector Production: Insights Into PiggyBac Transposase and Concatemeric Array Strategies
Source: Biotechnol J. 2025 Oct 6;20(10):e70135. doi: 10.1002/biot.70135 (PMC12501403; doi:10.1002/biot.70135)

Supplementary Figure Legends

Supplementary Figure 1: **Correlation analysis.** Comparisons of absolute infectious titer with IVCC for the whole LVV production period (A), specific growthrate µ between 168 and 192 h post induction (B) and whole LVV production period (C), viability 192 h post induction (D), amount of selection marker (E), and total DNA transfected (F).

L3 = Lipofectamine 3000, L2 = Lipofectamine 2000, Zeo20/40/50/100 = Zeocin concentration (µg/ml), 12.2 -- 62.5 µg = total DNA used, # = replicate, neg. C. = negative Control, linear. = linearized transposon plasmid, 5:1/10:1/20:1 = ratio transposon vector to transposase vector.


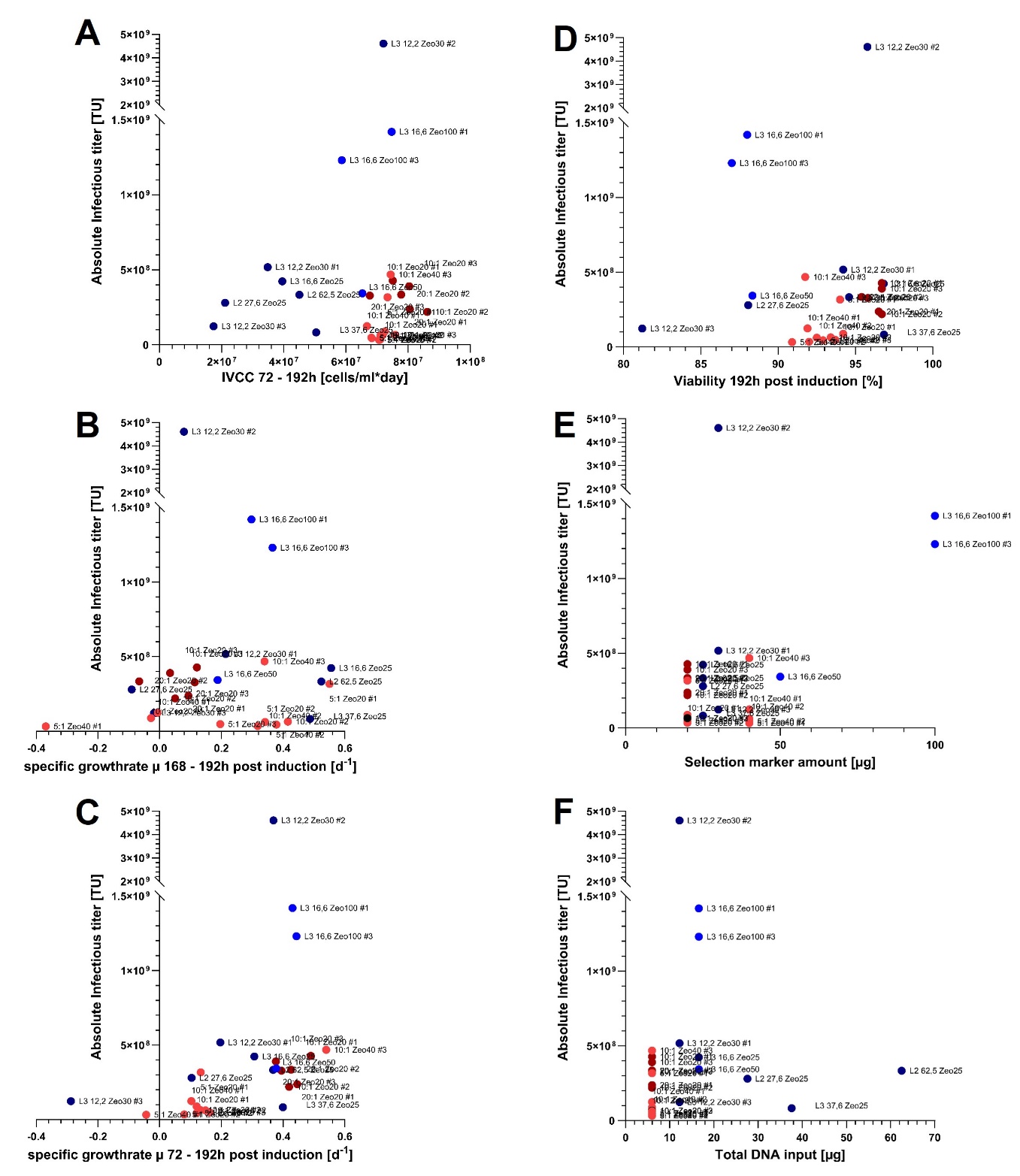

Supplement: Supplementary file 1 — Supporting Information file 1: biot70135‐sup‐0001‐FigureS1.docx [file BIOT-20-e70135-s001.docx]
